# SHED aggregate exosomes shuttled miR‐26a promote angiogenesis in pulp regeneration via TGF‐β/SMAD2/3 signalling

**DOI:** 10.1111/cpr.13074

**Published:** 2021-06-07

**Authors:** Meiling Wu, Xuemei Liu, Zihan Li, Xiaoyao Huang, Hao Guo, Xiaohe Guo, Xiaoxue Yang, Bei Li, Kun Xuan, Yan Jin

**Affiliations:** ^1^ State Key Laboratory of Military Stomatology & National Clinical Research Center for Oral Diseases & Shaanxi Clinical Research Center for Oral Diseases Department of Preventive Dentistry School of Stomatology The Fourth Military Medical University Xi'an China; ^2^ State Key Laboratory of Military Stomatology & National Clinical Research Center for Oral Diseases & Shaanxi International Joint Research Center for Oral Diseases Center for Tissue Engineering School of Stomatology The Fourth Military Medical University Xi'an China; ^3^ Liaoning Provincial Key Laboratory of Oral Diseases Department of Pediatric Dentistry School and Hospital of Stomatology China Medical University Shenyang China

**Keywords:** angiogenesis, exosome, miR‐26a, pulp regeneration, SHED aggregate, TGF‐β signalling

## Abstract

**Objectives:**

Pulp regeneration brings big challenges for clinicians, and vascularization is considered as its determining factor. We previously accomplished pulp regeneration with autologous stem cells from deciduous teeth (SHED) aggregates implantation in teenager patients, however, the underlying mechanism needs to be clarified for regenerating pulp in adults. Serving as an important effector of mesenchymal stem cells (MSCs), exosomes have been reported to promote angiogenesis and tissue regeneration effectively. Here, we aimed to investigate the role of SHED aggregate‐derived exosomes (SA‐Exo) in the angiogenesis of pulp regeneration.

**Materials and Methods:**

We extracted exosomes from SHED aggregates and utilized them in the pulp regeneration animal model. The pro‐angiogenetic effects of SA‐Exo on SHED and human umbilical vein endothelial cells (HUVECs) were evaluated. The related mechanisms were further investigated.

**Results:**

We firstly found that SA‐Exo significantly improved pulp tissue regeneration and angiogenesis in vivo. Next, we found that SA‐Exo promoted SHED endothelial differentiation and enhanced the angiogenic ability of HUVECs, as indicated by the in vitro tube formation assay. Mechanistically, miR‐26a, which is enriched in SA‐Exo, improved angiogenesis both in SHED and HUVECs *via* regulating TGF‐β/SMAD2/3 signalling.

**Conclusions:**

In summary, these data reveal that SA‐Exo shuttled miR‐26a promotes angiogenesis via TGF‐β/SMAD2/3 signalling contributing to SHED aggregate‐based pulp tissue regeneration. These novel insights into SA‐Exo may facilitate the development of new strategies for pulp regeneration.

## Graphical abstract

The underlying mechanisms of the SHED aggregate involved in pulp regeneration is revealed in this paper. SHED aggregate‐derived exosomes (SA‐Exo) shuttled miR‐26a promote SHED endothelial differentiation and enhance the angiogenic ability of HUVECs via TGF‐β/SMAD2/3 signalling, which contributing to angiogenesis in pulp tissue regeneration.

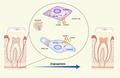

## 1 INTRODUCTION

Dental pulp, a highly organized soft tissue situated in a confined environment surrounded by dentin walls, plays a seminal role in maintaining tooth homeostasis.^1^ However, the dental pulp is prone to irreversible pulpitis or necrosis resulting from dental traumas and infections.^2^ The traditional therapeutic intervention, known as root canal therapy, is based on infected dental pulp extirpation and bioinert material root canal filling resulting in a devitalized tooth, which is more vulnerable to reinfections and fractures.^3^ In recent years, the development of stem cell‐based regenerative medicine has shown immense potential for tooth viability restoration and pulp regeneration.^4^ Nevertheless, functional pulp regeneration remains challenging.

We have previously applied autologous SHED aggregate to animals and patients with immature permanent teeth. The regeneration of dental pulp containing an odontoblast layer, blood vessels and nerves, the rescue of sensation to stimuli and continued root development have been successfully achieved.^5^ However, other studies have attempted to employ autologous mobilized dental pulp stem cells or allogeneic umbilical cord mesenchymal stem cells in mature devitalized teeth, even with a favourable clinical efficacy, blood perfusion and sensitivity restoration are not satisfactory.^6^, ^7^ One of the most primary factors for this is the unique anatomy of pulp space, which is an inextensible environment allowing only a single blood supply from its small apical canal opening.^8^ While securing an adequate blood supply is a determining element of success for pulp regeneration.^9^ Therefore, the detailed mechanisms, particularly how to rebuild blood supply in regenerated dental pulp, should be elucidated.

It has been indicated that vascularization during pulp regeneration derives not only from the invading host blood vessels but also the differentiation of transplanted stem cells into the endothelial cells forming vessels,^8^, ^9^, ^10^ whereas the exact role of transplanted SHED aggregates participate in angiogenesis remains elusive. It has been known that SHED possess highly proliferative and multilineage differentiation potential, including direct endothelial differentiation.^11^, ^12^ Moreover, paracrine of what from SHED has been proved to activate HUVECs and promote all processes of angiogenesis.^13^ Exosomes have been regarded as nanometre‐sized membranous extracellular vesicles, which are enriched with bioactive biomolecules (DNA, RNA, lipids, metabolites and proteins, for instance) capable of transducing cell‐to‐cell communication in a paracrine or autocrine manner.^14^, ^15^, ^16^, ^17^ In their multifaceted role of modulating biological responses, the involvement of exosomes in angiogenesis has been documented and their therapeutic application potential in regeneration is increasingly being concerned.^18^, ^19^ Although previous study has demonstrated that dental pulp cell‐derived exosomes possess angiogenic potential with possible therapeutic effects in regenerative endodontics.^20^ Whether exosomes derived from transplanted SHED aggregates promote angiogenesis in pulp regeneration and the underlying mechanisms remain unclear, which urgently requires further exploration.

In this work, we first utilized immunodeficient mice model to evaluate the efficacy of SA‐Exo in pulp regeneration. We found that SA‐Exo significantly improved angiogenesis in pulp tissue regeneration. Then, we investigated the role of SA‐Exo in the angiogenic potential of SHED and HUVECs. We found that SA‐Exo promoted SHED endothelial differentiation and enhanced angiogenesis of HUVECs. Mechanistically, we revealed that miR‐26a transferred by SA‐Exo promoted SHED and HUVECs angiogenesis via TGF‐β/SMAD2/3 signalling. Collectively, exosomes secreted from SHED aggregates would facilitate vascularization, which partially unravelled the underlying mechanisms of pulp regeneration with SHED aggregates implantation and provided a potential way to further improve pulp regeneration outcomes.

## 2 MATERIALS AND METHODS

### 2.1 Exosome extraction and characterization

SHED were isolated and SHED aggregates were prepared as described previously.^5^ Nearly 3 × 10^7^ P2 SHED were seeded in the 10‐layer cell factory (Thermo Nunc, United States). When the cell culture reached 80% confluence, 10% alpha modification of Eagle's medium (α‐MEM; Gibco, United States) containing VC (30 mg/mL) was replaced. Cells were induced to form lamellar sheets about 7‐14 days. Then, the SHED aggregate or SHED were cultured in the α‐MEM without foetal bovine serum (FBS; Gibco) for another 72 hours. Finally, the medium was collected and exosomes were extracted using ultracentrifugation. Briefly, the supernatant was firstly centrifuged at 2000 g for 10 minutes. Next, the supernatant was centrifuged at 10 000 g for 30 minutes. The supernatant was then ultracentrifuged at 100 000 *g* for 70 minutes. Finally, the pellet was washed using phosphate‐buffered saline by ultracentrifugation at 100 000 g for 70 minutes again. Transmission electron microscopy (TEM) (Thermo Fisher, United States) was used to observe the morphology of exosomes. Nanoparticle tracking analysis (NTA) was used to determine the size of exosomes. Western blotting was used to identify the expression of CD9 (ab92726, Abcam, United Kingdom), CD63 (ab217345, Abcam), CD81 (ab109201, Abcam) and TSG101 (ab125011, Abcam) of exosomes. SHED‐derived exosomes were used as a standard control.

### 2.2 Exosomes inhibition

SHED aggregates were prepared with conditioned medium supplemented with or without 20 μmol/L GW4869 (HY‐19363, MedChemExpress, United States). After 10 days, the SHED aggregates were collected for implantation. SHED were pre‐incubated with or without 20 μmol/L GW4869 for 48 hours, and the effect of inhibition was measured using the bicinchoninic acid assay (BCA) (Beyotime, China).

### 2.3 Animal experiments

The animal experiments were approved by the Institutional Animal Care and Use Committee of the Fourth Military Medical University (No. IACUC‐20200401). Immunodeficient mice of 6‐week‐old were purchased from Hunan SJA Laboratory Animal Co., Ltd. (Hunan, China). The mice were kept under specific pathogen‐free conditions with free access to food and water.

The mice were distributed into four groups: GW4869 group (SHED aggregate pre‐treated with GW4869), SHED group (regularly prepared SHED aggregate), Exosomes group (regularly prepared SHED aggregate+SA‐Exo) and GW4869+Exosomes group (SHED aggregate pre‐treated with GW4869+SA‐Exo). Cell aggregates with or without SA‐Exo were introduced into the tooth fragments and transplanted subcutaneously into the backs of the mice. At 12 weeks post‐implantation, the mice were sacrificed and the tooth fragments were collected and fixed in 4% paraformaldehyde for histological analysis.

### 2.4 Histological analysis

After 24 hours fixation, the tooth fragments were decalcified using 17% ethylene diamine tetra acetic acid (EDTA) (MP Biomedicals, United States) and then embedded in paraffin. The 4‐μm‐thick sections were obtained and dehydrated with graded ethanol. Haematoxylin and eosin (H&E) staining was carried out using a commercial staining kit according to the manufacturer's instructions. Photographs were taken using a microscope (OLYMPUS, Japan).

### 2.5 Immunofluorescence analysis

After decalcification, the tooth fragments were dehydrated with 30% saccharose, embedded into the optimal temperature compound (OCT) (Leica, United States), and 10 μm thick sections were obtained. Next, the sections were permeabilized with 0.05% Triton X‐100 (Sigma‐Aldrich, United States) and subsequently blocked using 5% bovine serum albumin (BSA) (MP Biomedicals). Next, the sections were incubated with the primary antibodies overnight at 4℃ and then incubated with the related fluorescence secondary antibodies at room temperature for 1 hour. DAPI was used for nuclei counterstaining. Images were captured using a confocal microscope (Nikon, Japan). Antibodies against CD31 (1:100, FAB3628G, R&D Systems, United States) and anti‐mitochondria antibody (1:100, ab92824, Abcam) were used in this study.

### 2.6 Exosome uptake assay

SHED or HUVECs were plated onto dishes in 10% FBS‐supplemented α‐MEM or Dulbecco's modified Eagle's medium (DMEM; Gibco) and cultured for 6 hours. SA‐Exo were labelled with the PKH67 Green Fluorescent Cell Linker Kit (Sigma‐Aldrich, United States) according to the manufacturer's instructions and then were co‐cultured with SHED or HUVECs for 2 hours. Subsequently, the cells were fixed with 4% paraformaldehyde. Cytoskeleton staining was performed using the CellMaskTM Deep Red Plasma Membrane Stain (Invitrogen, United States) and the nuclei were counterstained with DAPI. Finally, the uptake phenomenon was observed using a laser scanning confocal microscope (Nikon, Japan).

### 2.7 Tube formation assay

Matrigel Matrix Basement membrane (Corning, United States) was dissolved and 96‐well plates were coated. SHED or HUVECs were seeded onto Matrigel‐coated wells and cultured in 1% FBS‐supplemented α‐MEM or DMEM in the presence of SA‐Exo/S‐Exo or the conditional medium. Tube formation was recorded using an inverted microscope (Leica, Germany). The network structures were calculated by selecting five random fields per well using ImageJ software (National Institutes of Health, USA).

### 2.8 Transwell assay

See Appendix 1.

### 2.9 Quantitative real‐time polymerase chain reaction (qRT‐PCR)

See Appendix 1.

### 2.10 Western blot analysis

See Appendix 1.

### 2.11 MiRNAs mimics and inhibitors in vitro

Hsa‐miR‐26a‐5p‐mimic and inhibitor were purchased from Ribo (Guangzhou, China). Cells were transfected with 50 nmol/L miRNA mimics, 100 nmol/L miRNA inhibitors and the corresponding concentration of negative control (cel‐miR‐39‐3p) using a riboFect CP Transfection Kit according to the manufacturer's instructions. Then, they were seeded onto six‐well plates and cultured for 48 hours at 37℃. For quantification, total RNA including miRNA was isolated using the miRNeasy Micro Kit (1071023, Qiagen, Germany). Expression levels of miR‐26a in exosomes were analysed using qRT‐PCR.

### 2.12 Small RNA extraction, library preparation and small RNA sequencing

Small RNA was extracted according to the manufacturer's instructions (Qiagen). Small RNA sequencing was carried out by BGISEQ‐2000 and the sequencing libraries were constructed using the BGI Online platform (Shenzhen, China). The raw data were converted to FASTQ format. Read quality was assessed by DNBseq to filter out reads of low quality, joint contamination and high unknown base content. MiRNAs with significant differences in expression between SHED and SHED aggregate were shown using the heat map based on the databases of miRbase. The KEGG pathway enrichment analyses were carried out and differentially expressed miRNAs were enriched in signalling pathways. The mentioned miRNA information of SA‐Exo and S‐Exo was available online (https://biosys.bgi.com/#/report/login).

### 2.13 Statistical analysis

Data are expressed as mean ± standard deviation (SD). Comparisons between two groups were performed using independent two‐tailed Student's *t* tests, and multiple group comparisons were performed by one‐way analysis of variance (ANOVA). *P* values < .05 were considered statistically significant. SPSS software (IBM, 19.0) and GraphPad Prism (GraphPad Software, 8.0) were used to perform statistical analysis and Graphs.

## 3 RESULTS

### 3.1 SA‐exo promote the dentine‐pulp complex regeneration and angiogenesis

**FIGURE 1:**
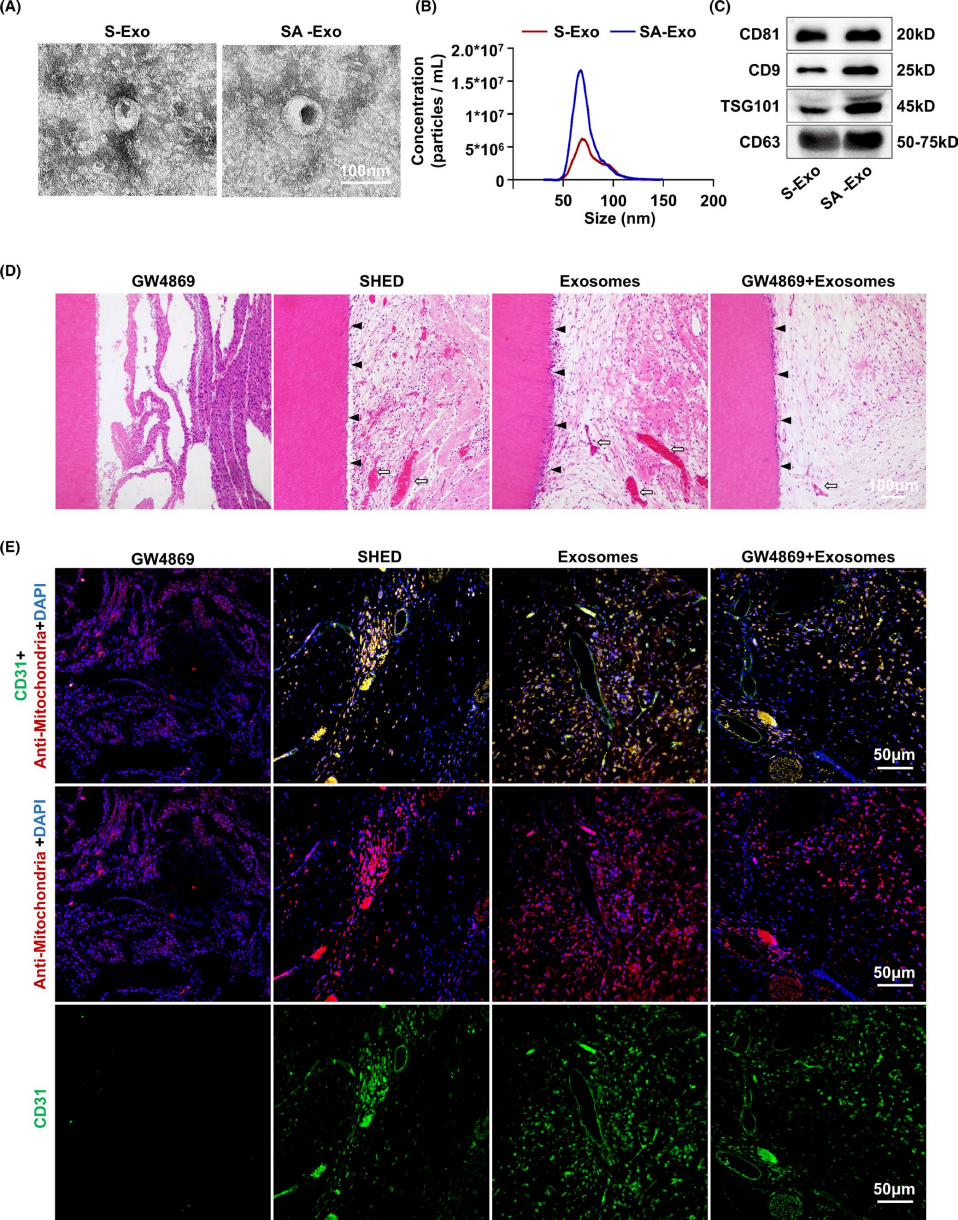
SA‐Exo promote the dentine‐pulp complex regeneration and angiogenesis. A, Transmission electron microscopy shows the typical morphology of SA‐Exo and S‐Exo. Scale bar, 100 nm. B, Nanoparticle tracking analysis (NTA) shows diameters of SA‐Exo and S‐Exo with approximately 50‐100 nm. C, Western blot analysis shows expression of CD9, CD63, CD81, and TSG 101 in SA‐Exo and S‐Exo. D, Haematoxylin and eosin **(**H&E) shows the formation of new dentin and pulp tissue with vessels when SHED aggregates are subcutaneously implanted into immunodeficient mice. The black triangle indicates odontoblast layer; the white arrow indicates vessles. n = 5. Scale bar, 100 μm. E, Immunofluorescence analysis shows the formation of new CD31 positive vessels. Scale bar, 50 μm. SA‐Exo, SHED aggregate exosomes. S‐Exo, SHED exosomes. GW4869, SHED aggregate pre‐treated with GW4869. SHED, SHED aggregate. Exosomes, SHED aggregate+SA‐Exo. GW4869+Exosomes, SHED aggregate pre‐treated with GW4869+SA‐Exo

**FIGURE A1:**
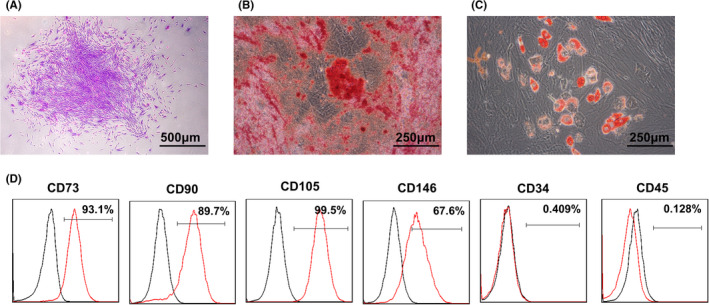
Characterization of SHED. A, SHED cultured in vitro exhibit spindle‐shaped morphology. Scale bar, 500 μm. B, Cultured SHED form mineralized nodules after osteogenic induction for 28 days. Scale bar, 250 μm. C, SHED cultured in adipogenic inductive condition for 21 days, lipid droplets are found by Oil Red O staining. Scale bar, 250 μm. D, Flow cytometric analysis reveals positive expression of CD73, CD90, CD105 and CD146, and negative expression of CD34, and CD45

Firstly, SHED were successfully isolated with typical characteristics of MSCs (Figure A1). Then, SHED aggregates were prepared, and SA‐Exo/S‐Exo were isolated and characterized by TEM analysis. SA‐Exo exhibited a typical morphology with a sphere‐like bilayer membrane structure, which were similar to S‐Exo (Figure 1A). Moreover, the NTA results showed that the mean diameters of SA‐Exo and S‐Exo were approximately 50‐100 nm (Figure 1B). Additionally, western blot analysis revealed the presence of exosome markers, including CD81, CD9, TSG101 and CD63, on both SA‐Exo and S‐Exo (Figure 1C).

**FIGURE A2:**
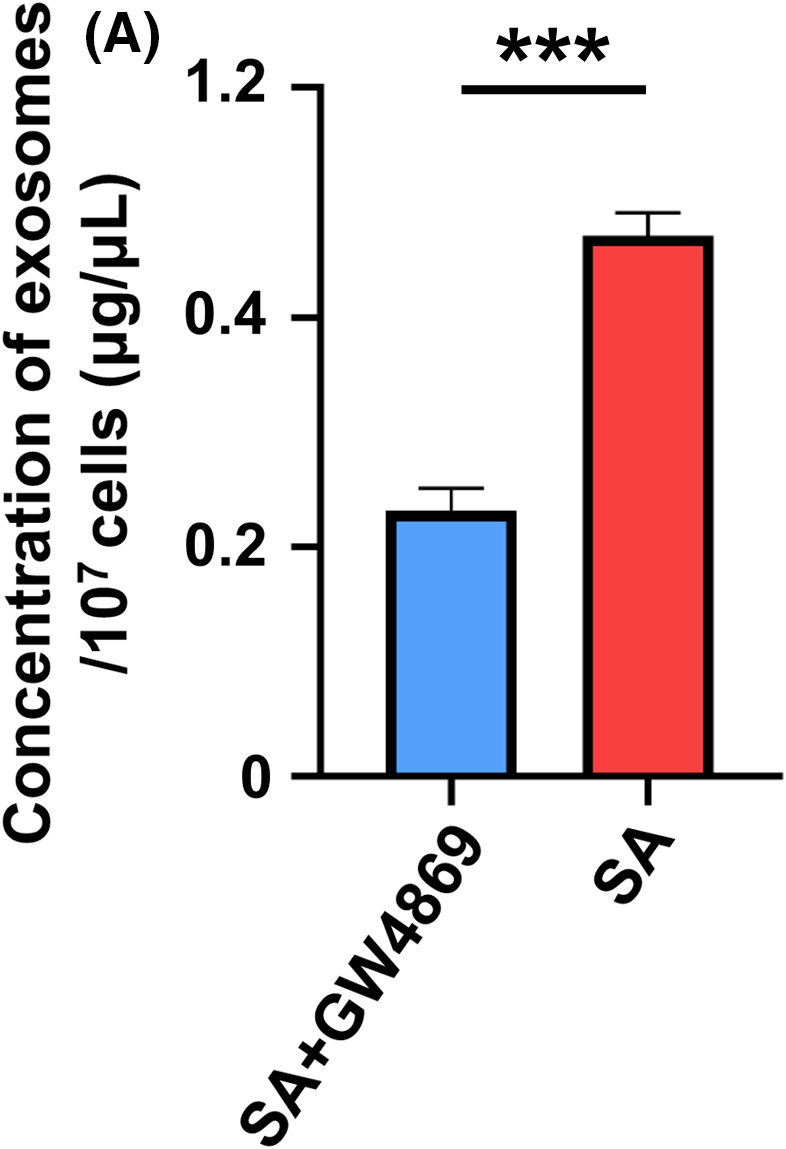
GW4869 inhibits exosome secretion in SHED aggregate. The bicinchoninic acid assay (BCA) shows the protein concentration of released exosomes of SHED aggregate treated with or without GW4869

To investigate the role of SA‐Exo in pulp regeneration, we inhibited the secretion of exosomes from SHED aggregates with GW4869 (Figure A2). Then, we implanted tooth fragments filled with SHED aggregate (SHED), SHED aggregate pre‐treated with GW4869 (GW4869), SHED aggregate combined with SA‐Exo (Exosomes), and GW4869 pre‐treated SHED aggregate supplemented with SA‐Exo (GW4869+Exosomes) subcutaneously into immunodeficient mice. Histological analysis showed that the dentin‐pulp complex was regenerated, which contained a new continuous dentine layer and newly formed blood vessels after SHED aggregate implantation (Figure 1D). Moreover, dentin‐pulp complex regeneration was enhanced with SHED aggregate and SA‐Exo combined implantation. However, regeneration was suppressed after SA‐Exo inhibition, which, in turn, was rescued with exogenous SA‐Exo supplementation (Figure 1D). Concomitantly, since vascularization is the critical factor for successful pulp regeneration, we detected the expression level of the angiogenic marker CD31 using immunofluorescence staining in the regenerated pulp tissue. The fluorescence intensity was higher in the SHED aggregate and SA‐Exo combined implantation than in SHED aggregate implantation. Less CD31 positive expression was observed in GW4869 pre‐treated SHED aggregate implantation, which was rescued by exogenous SA‐Exo supplementation (Figure 1E). Double‐stained CD31 and human mitochondria cells were observed in the SHED, Exosomes and GW4869+Exosomes groups (Figure 1E). Collectively, these results indicated that SA‐Exo promoted the dentine‐pulp complex regeneration and angiogenesis in SHED aggregate‐based dental pulp regeneration. Moreover, blood vessels in the regenerated pulp originated from implanted SHED and host cells.

### 3.2 SA‐exo enhance SHED endothelial differentiation

**FIGURE 2:**
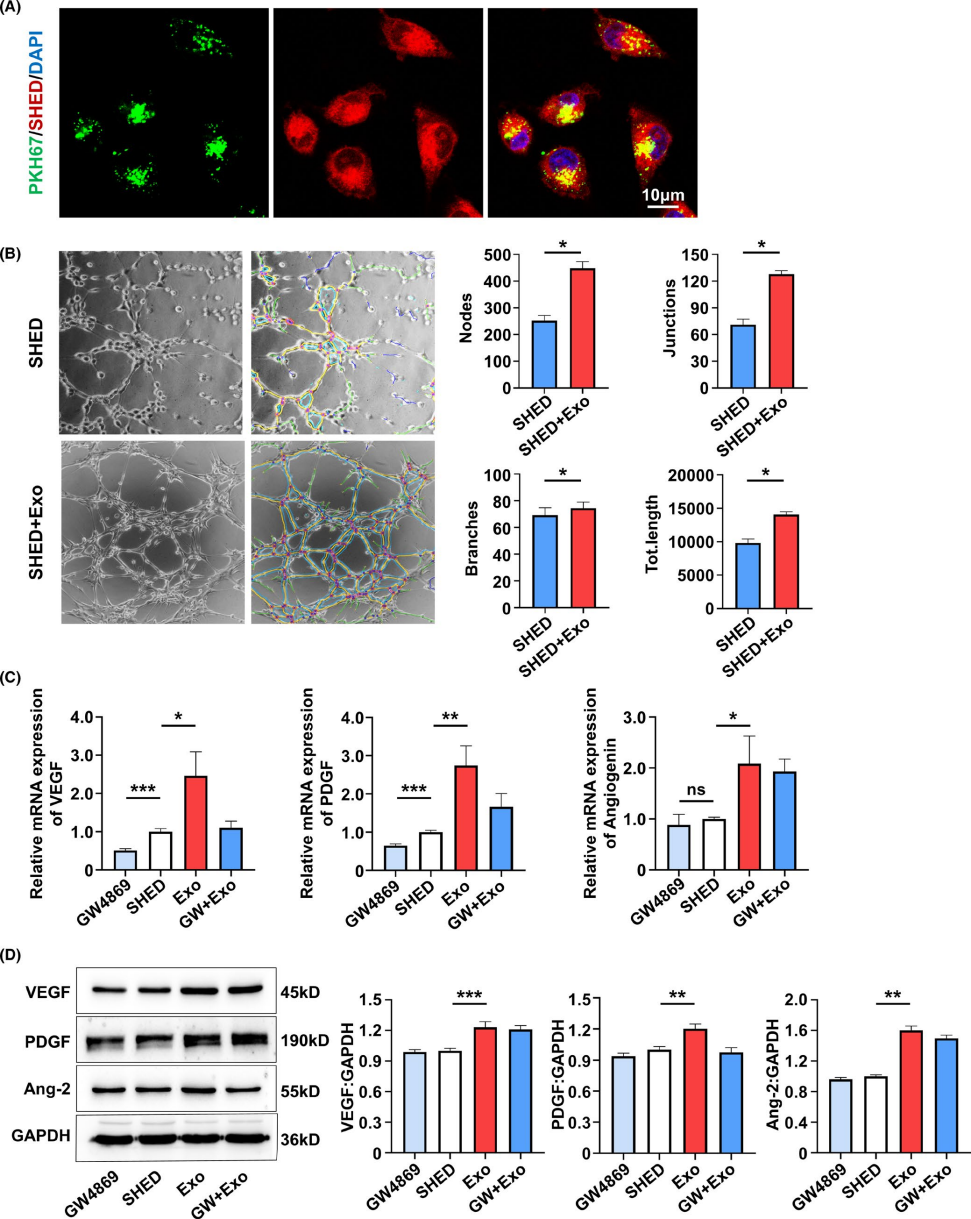
SA‐Exo enhance endothelial differentiation of SHED. A, Immunofluorescence analysis shows uptake of SA‐Exo by SHED. Scale bar, 10 μm. B, Tube formation analysis shows endothelial differentiation of SHED. C, Quantitative real‐time polymerase chain reaction (qRT‐PCR) shows the expression of *VEGF*, *PDGF* and *angiogenin* in SHED. D, Western blot analysis shows VEGF, PDGF and angiopoietin 2 expression in SHED. All results are generated in three independent experiments. Data are shown as mean ± standard deviation (SD); *P < .05; **P < .01; ***P < .001. GW4869, SHED pre‐treated with GW4869. Exo, SHED+SA‐Exo. GW+Exo, SHED pre‐treated with GW4869+SA‐Exo

**FIGURE A3:**
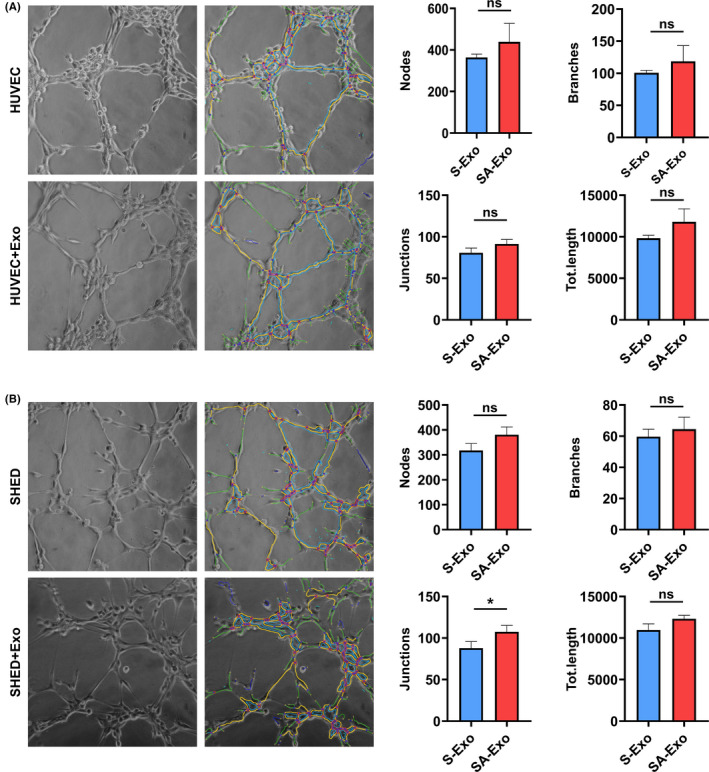
SA‐Exo enhance the angiogenic potential of SHED and HUVECs compared to S‐Exo without statistical significance. A, Tube formation analysis shows endothelial differentiation of SHED. B, Tube formation analysis shows the tube formation capacity of HUVECs

Our results and previous studies showed that the newly formed vessels in pulp regeneration are derived from both transplanted cells and host cells.^8^, ^9^ To assess whether SA‐Exo contribute to endothelial differentiation of SHED, we examined the angiogenic function of SHED after exogenous SA‐Exo treatment. First, we found that PKH67‐labelled SA‐Exo were internalized by SHED (Figure 2A). Then, the tube formation of SHED was evaluated and the results showed that the total number of nodes, junctions, branches and length were significantly increased in the coculture group compared to the SHED group (Figure 2B). While, SA‐Exo enhanced the tube formation of SHED compared to S‐Exo without statistic difference (Figure A3(A)). Moreover, we analysed the expression of angiogenesis‐associated genes using qRT‐PCR after SHED and SA‐Exo coculture. The expression levels of *VEGF*, *angiogenin* and *PDGF* of SHED were significantly upregulated after SA‐Exo treatment. However, the expression of *VEGF* and *PDGF* were downregulated after GW4869 treatment, while, which were partially rescued by the additional SA‐Exo application (Figure 2C). As shown by western blotting, the expression of the angiogenesis‐associated protein (VEGF, angiopoietin 2 and PDGF) of SHED was upregulated by SA‐Exo. These proteins were downregulated by GW4869 treatment compared to the SHED group, however, there was no statistic difference (Figure 2D). Therefore, these data demonstrated that SA‐Exo can directly enhance the endothelial differentiation potential of SHED, which would promote pulp regeneration.

### 3.3 SA‐exo upregulate the angiogenic ability of HUVECs

**FIGURE 3:**
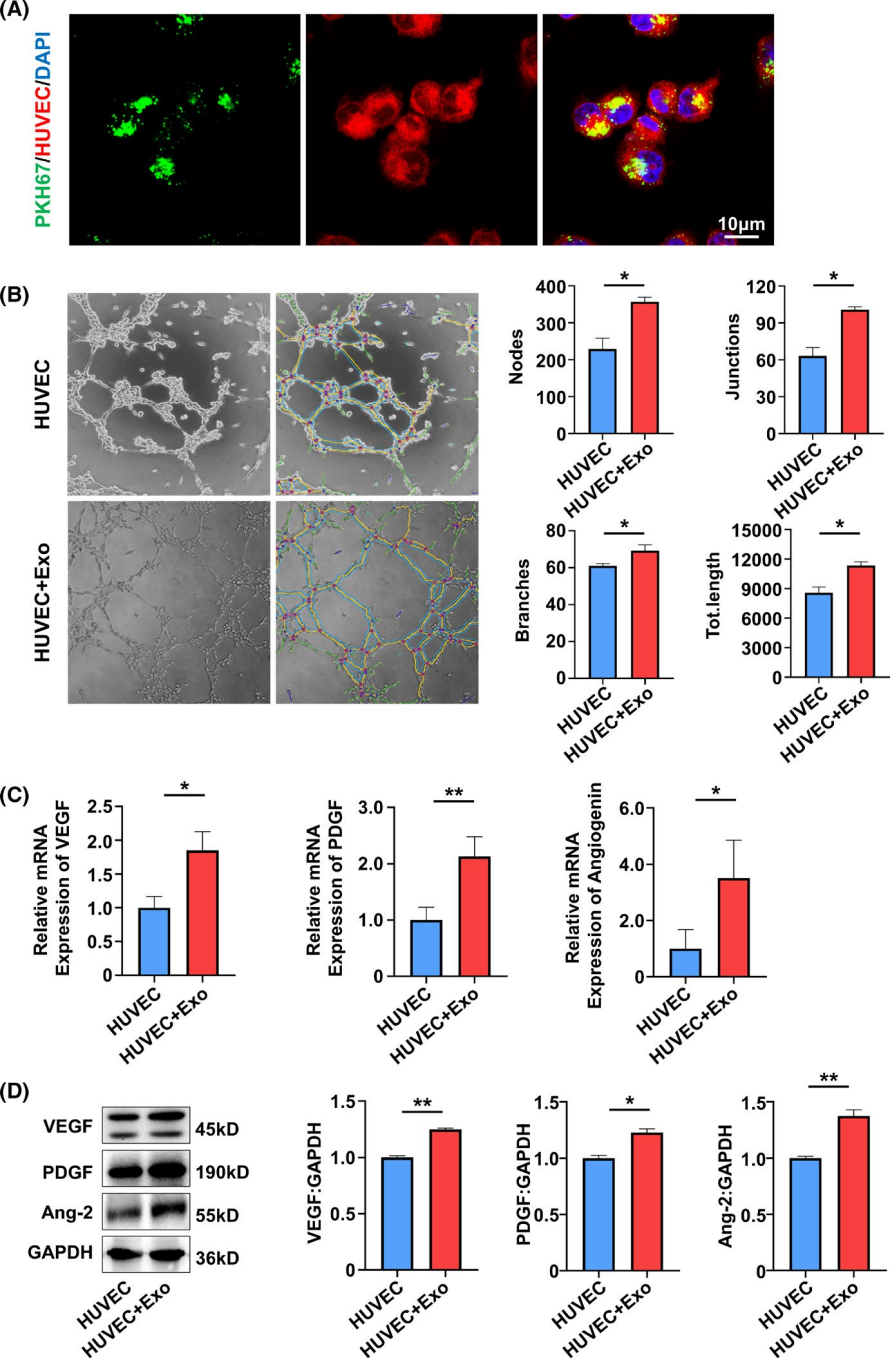
SA‐Exo upregulate the angiogenic ability of HUVECs. A, Immunofluorescence analysis shows uptake of SA‐Exo by HUVECs. Scale bar, 10 μm. B, Tube formation analysis shows the tube formation capacity of HUVECs. C, Quantitative real‐time polymerase chain reaction (qRT‐PCR) shows the expression of *VEGF, PDGF,* and *angiogenin* in HUVECs. D, Western blot analysis shows VEGF, PDGF and angiopoietin 2 expression in HUVECs. All results are generated in three independent experiments. Data are shown as mean ± standard deviation (SD); *P < .05; **P < .01

**FIGURE A4:**
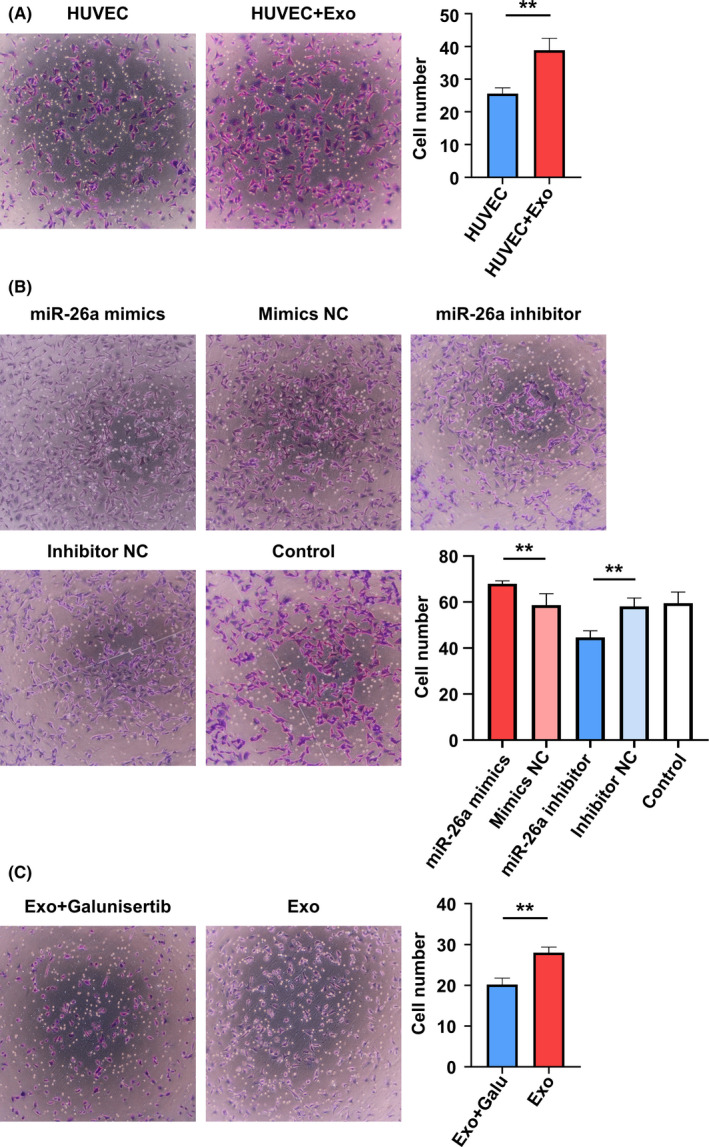
SA‐Exo promote HUVECs migration. A, Cell migration analysis shows the migration ability of HUVECs after SA‐Exo treatment. B, Cell migration analysis shows the migration ability of HUVECs after miR‐26a regulated. C, Cell migration analysis shows the migration ability of HUVECs treated with SA‐Exo and TGFβ RI inhibition

Endothelial cells are the other important cells in pulp angiogenesis and regeneration. To further determine the effect of SA‐Exo on the angiogenic ability of endothelial cells, SA‐Exo and HUVECs were cocultured for further assessment. It was observed that SA‐Exo were internalized by HUVECs similar to SHED (Figure 3A). Next, the tube formation assay showed that SA‐Exo significantly increased the number of nodes, junctions, branches and total tube length in network structures of HUVECs compared with those without SA‐Exo (Figure 3B). Also, the tube formation of HUVECs was increased by SA‐Exo compared to S‐Exo with no statistical significance (Figure A3(B)). Furthermore, as indicated by the transwell assay, the migration of HUVECs was also significantly enhanced after cocultured with SA‐Exo (Figure A4(A)). The angiogenesis‐associated genes *VEGF*, *angiogenin* and *PDGF* detected by qRT‐PCR were upregulated after HUVECs were cocultured with SA‐Exo (Figure 3C). Additionally, the expression levels of angiogenesis‐associated protein of HUVECs were also increased by SA‐Exo treatment (Figure 3D). Collectively, these results indicated that SA‐Exo upregulate the angiogenic ability of HUVECs, which contributes to pulp regeneration.

### 3.4 MiR‐26a contributes to SA‐exo‐mediated endothelial differentiation of SHED

**FIGURE 4:**
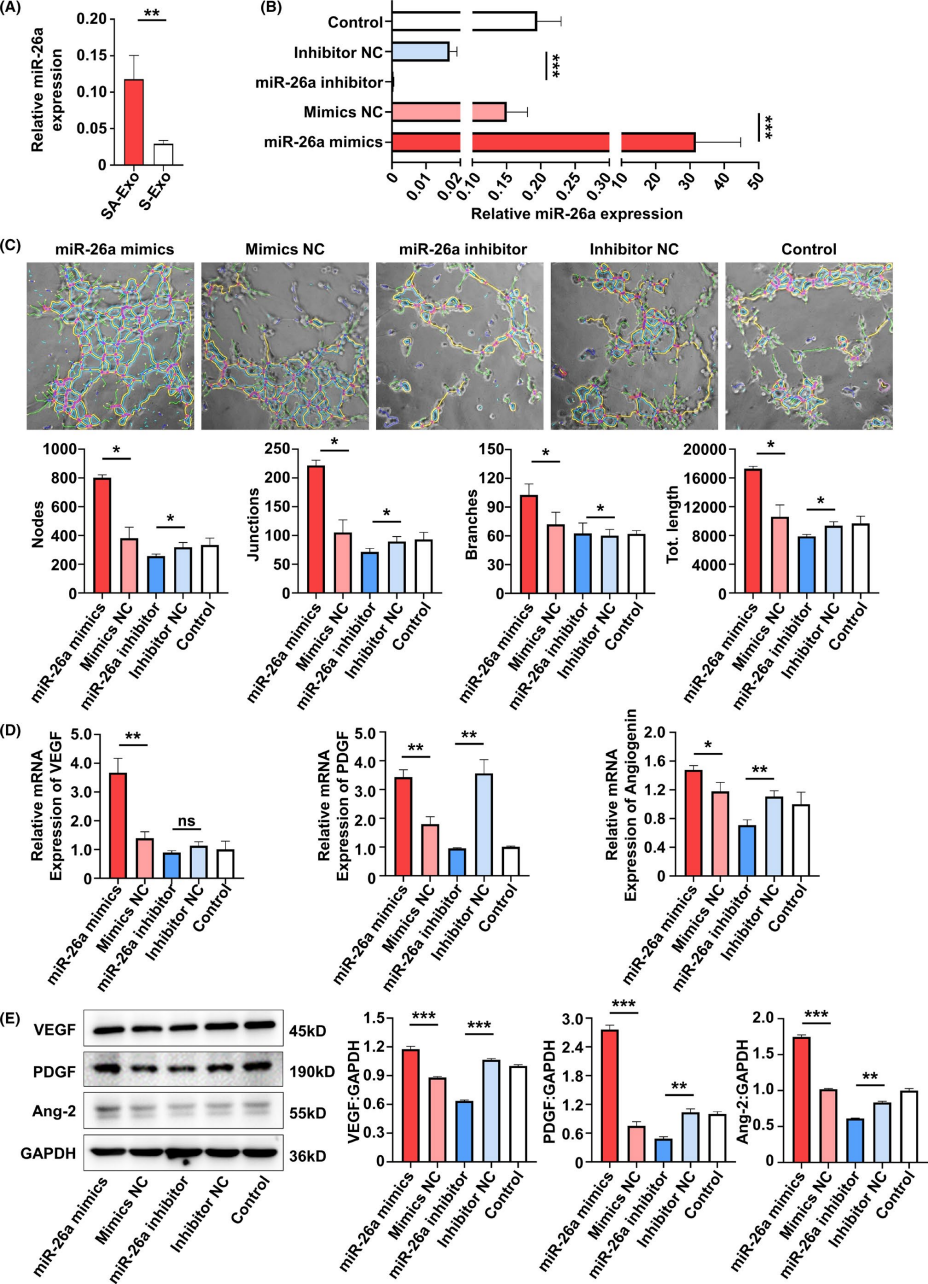
MiR‐26a contributes to SA‐Exo‐mediated endothelial differentiation of SHED. A, Quantitative real‐time polymerase chain reaction (qRT‐PCR) shows the expression of miR‐26a in SA‐Exo and S‐Exo. B, qRT‐PCR shows the expression of miR‐26a in SA‐Exo. C, Tube formation analysis shows endothelial differentiation of SHED. D, qRT‐PCR shows the expression of *VEGF*, *angiogenin* and *PDGF* in SHED. E, Western blot analysis shows VEGF, Angiopoietin 2 and PDGF expression in SHED. All results are generated in three independent experiments. Data are shown as mean ± standard deviation (SD); ns, not significant; *P < .05; **P < .01; ***P < .001

**FIGURE A5:**
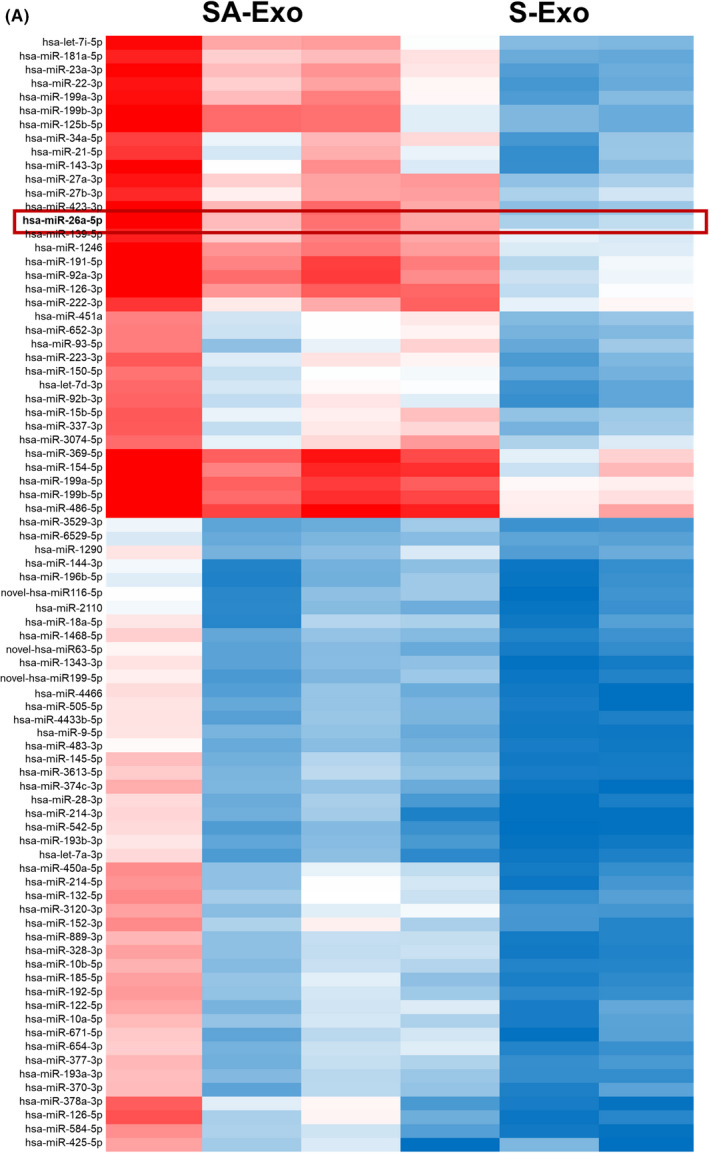
MiR‐26a is enriched in SA‐Exo. Heat map of miRNA expression shows miR‐26a is upregulated in SA‐Exo

Exosomes elicit biological activity by delivering their cargos to the recipient cells.^21^ MSC exosomal miRNAs have been implicated important in many MSC exosome‐mediated cellular activities.^22^ Therefore, to gain insights into how the angiogenesis of SHED and HUVECs were promoted by SA‐Exo, we analysed the miRNA expression profiles of SA‐Exo. S‐Exo were used as a standard control. The heat map of miRNA expression demonstrated that SA‐Exo had a miRNA expression signature different from S‐Exo and miR‐26a was markedly upregulated in SA‐Exo (Figure A5). The qRT‐PCR result confirmed that the expression of miR‐26a was significantly higher in SA‐Exo (Figure 4A). Next, the effect of miR‐26a on the endothelial differentiation of SHED was investigated after upregulation or downregulation of miR‐26a, respectively. The expression of miR‐26a in SA‐Exo was significantly overexpressed by pre‐treating SHED aggregate with the miR‐26a mimics, in contrast, miR‐26a in SA‐Exo was suppressed by using the miR‐26a inhibitor (Figure 4B). The number of nodes, junctions, branches and total tube length increased in the miR‐26a mimics group compared to the miR‐26a mimics NC group. Additionally, the tube formation index decreased in the miR‐26a inhibitor group compared to the miR‐26a inhibitor NC group (Figure 4C). The genes expression was upregulated in the miR‐26a mimics group, and the expression of *PDGF* and *Angiogenin* was downregulated in the miR‐26a inhibitor group as shown by qRT‐PCR (Figure 4D). Furthermore, the expression of angiogenesis‐associated proteins were increased with miR‐26a upregulation and decreased with miR‐26a downregulation detected by western blotting (Figure 4E). These data demonstrated that miR‐26a could serve as a powerful bioactive component of SA‐Exo to promote SHED endothelial differentiation.

### 3.5 MiR‐26a contributes to SA‐exo‐mediated angiogenic improvement of HUVECs

**FIGURE 5:**
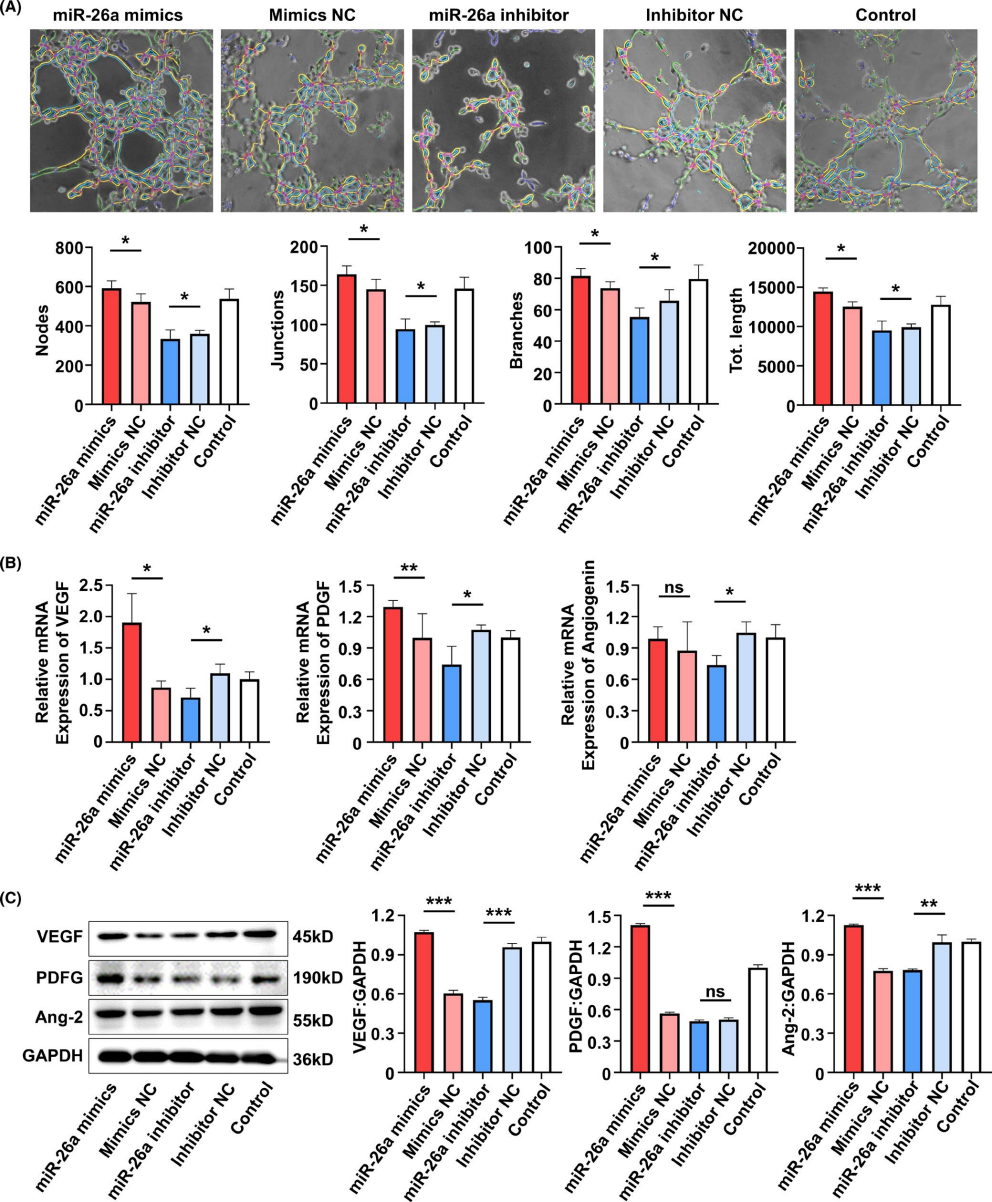
MiR‐26a contributes to SA‐Exo‐mediated angiogenic improvement of HUVECs. A, Tube formation analysis shows the tube formation capacity of HUVECs. B, Quantitative real‐time polymerase chain reaction (qRT‐PCR) shows the expression of *VEGF, PDGF* and *angiogenin* in HUVECs. C, Western blot shows VEGF, PDGF and angiopoietin 2 expression in HUVECs. All results are generated in three independent experiments. Data are shown as mean ± standard deviation (SD); ns, not significant; *P < .05; **P < .01; ***P < .001

The role of miR‐26a in the function of HUVECs were also detected. As shown by the tube formation assay, angiogenesis of HUVECs was enhanced in the miR‐26a mimics group compared to the miR‐26a mimics NC group. However, the functions were reduced after miR‐26a was inhibited (Figure 5A). Additionally, cell migration was increased in the miR‐26a mimics group compared to the miR‐26a mimics NC group, which was decreased in the miR‐26a inhibitor group (Figure A4(B)). Meanwhile, the expression of angiogenesis‐associated genes (*VEGF* and *PDGF*) was upregulated in the miR‐26a mimics group and the expression of *VEGF*, *PDGF* and *Angiogenin* was downregulated in the miR‐26a inhibitor group (Figure 5B). Similarly, the protein expression was increased in the miR‐26a mimics group, while the expression of VEGF and angiopoietin 2 was decreased in the miR‐26a inhibitor group (Figure 5C). These data indicated that the function of HUVECs can be improved by SA‐Exo *via* miR‐26a.

### 3.6 MiR‐26a promotes angiogenesis *via* upregulating TGF‐β/SMAD2/3 signalling

**FIGURE 6:**
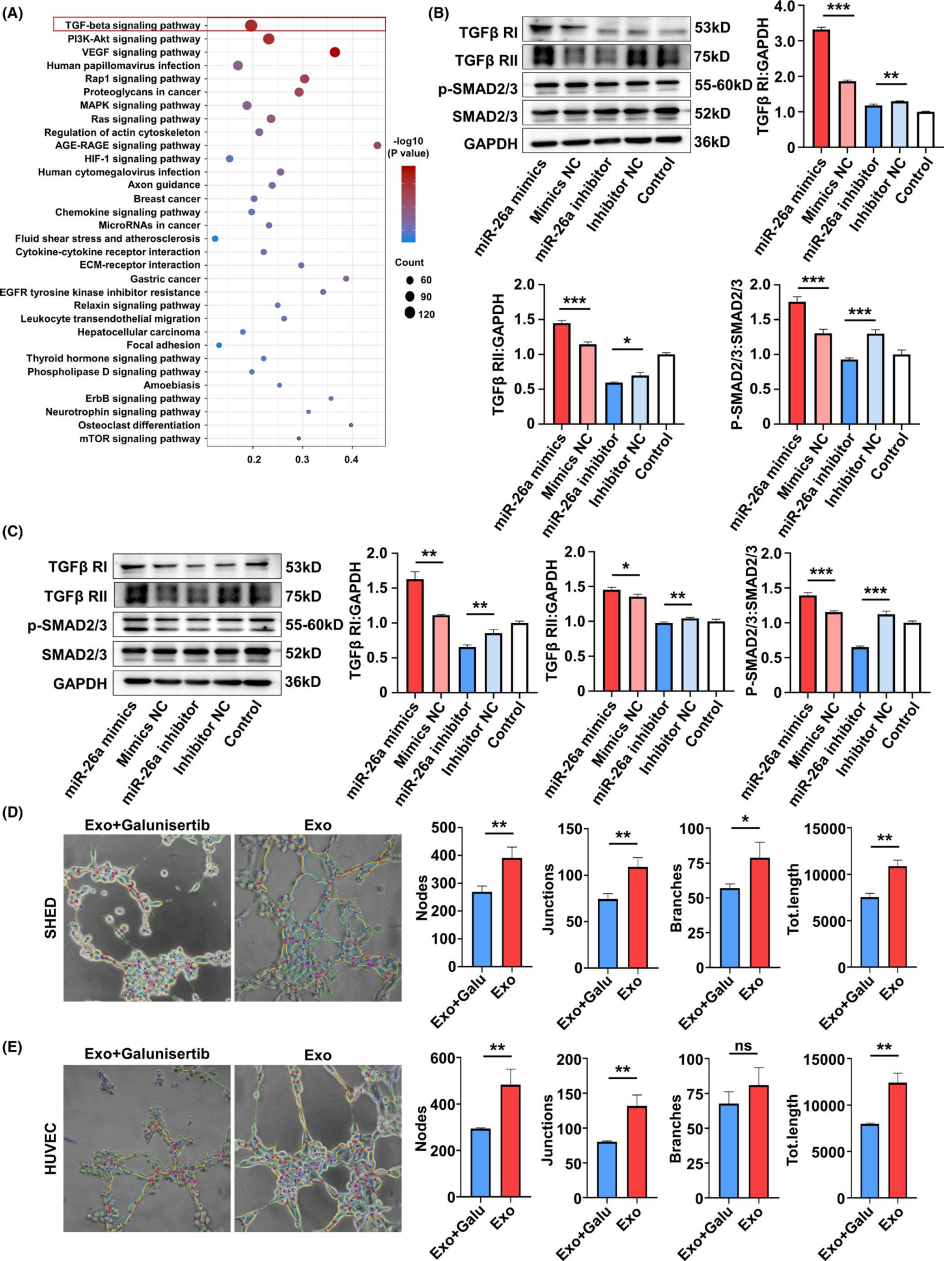
MiR‐26a promotes angiogenesis *via* upregulating TGF‐β/SMAD2/3 signalling. A, Pathway analysis shows TGF‐β signalling upregulated in SA‐Exo. B, Western blot analysis shows TGFβ RI, TGFβ RII, p‐SMAD2/3 and SMAD2/3 expression in SHED. C, Western blot analysis shows TGFβ RI, TGFβ RII, p‐SMAD2/3 and SMAD2/3 expression in HUVECs. D, Tube formation analysis shows the tube formation capacity of SHED. E, Tube formation analysis shows the tube formation capacity of HUVECs. All results are generated in three independent experiments. Data are shown as mean ± standard deviation (SD); ns, not significant; *P < .05; **P < .01; ***P < .001

**FIGURE A6:**
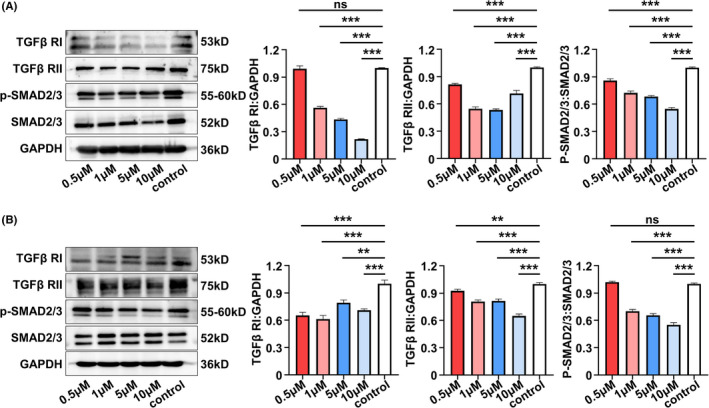
TGFβ RI serves as a key regulator of the TGF‐β signalling pathway. A, Western blot analysis shows TGFβ RI, TGFβ RII, p‐SMAD2/3 and SMAD2/3 expression in SHED. B, Western blot analysis shows TGFβ RI, TGFβ RII, p‐SMAD2/3 and SMAD2/3 expression in HUVECs

To further elucidate the detailed signalling pathways related to the proangiogenic role of miR‐26a, KEGG pathway analysis was conducted in SA‐Exo and S‐Exo. The results showed that of the multiple signal transductions involved in SA‐Exo activity, transforming growth factor‐beta (TGF‐β) signalling pathway was the most abundant (Figure 6A). To investigate whether miR‐26a targeted on TGF‐β signalling in SA‐Exo‐mediated angiogenesis facilitation, we used western blot analysis to assess SMAD2/3, p‐SMAD2/3, TGFβ RI and TGFβ RII protein levels during TGF‐β pathway activation in SHED and HUVECs. The results showed that overexpression of miR‐26a activated the TGF‐β pathway by upregulating p‐SMAD2/3, TGFβ RI and TGFβ RII in SHED and HUVECs compared to the miR‐26a mimics NC group. Furthermore, these TGF‐β pathway signals were downregulated after miR‐26a inhibition (Figure 6B,C). Accordingly, when the TGF‐β signalling pathway was inhibited by TGFβ RI inhibitor, Galunisertib (Figure A6(A,B)), the endothelial differentiation of SHED was decreased (Figure 6D). Moreover, the angiogenesis functions of HUVECs were also inhibited significantly by Galunisertib treatment (Figure 6E) and its migration capability (Figure A4(C)). Together, these findings demonstrated that SA‐Exo shuttled miR‐26a activated TGF‐β/SMAD2/3 signalling to promote SHED endothelial differentiation and improve endothelial cell function.

## 4 DISCUSSION

The emergence of regenerative endodontics is recognized as a prospective approach for tooth preservation and revitalization.^23^ In recent years, considerable efforts have been made to optimize the outcomes and extend understanding of regenerative endodontics.^24^, ^25^ Our previous clinical trial successfully achieved regeneration of pulp tissue equipped with blood vessels and sensory nerves by autologous SHED aggregate implantation into immature permanent teeth.^5^ However, the underlying mechanisms, especially vascularization which serve as the determinate element of pulp regeneration, have not been revealed yet. In the present study, our results demonstrated that SA‐Exo contributed to pulp tissue angiogenesis and regeneration, which relied on promoting SHED endothelial differentiation and enhancing the angiogenic ability of endothelial cells. Mechanistically, we found that SA‐Exo shuttled miR‐26a were in charge of facilitating angiogenesis *via* TGF‐β/SMAD2/3 signalling. These findings extend the current understanding of pulp regeneration, which might lead to a combinational use of SHED aggregates and exosomes, providing an alternative option for optimizing the therapeutic effect of regenerative endodontics.

Pulp regeneration is a complex process, and several key elements including stem cells, scaffolds and trophic factors, are finely orchestrated.^26^ However, enhancing neovascularization is still a challenge for achieving the complete vascularized and innervated pulp regeneration.^27^ The property of MSCs prepared in vitro depends on their origin, culture media and techniques.^28^ Cell sheet/aggregate techniques represent an important strategy for stem cell‐based regeneration, which could deliver high‐density stem cells and preserve the self‐produced tissue‐specific extracellular matrix. The extracellular matrix of cell sheet/aggregate techniques mimic natural microenvironments in terms of various mechanical, chemical and biological properties, which effectively guarantee the quality and biological properties of the transplanted stem cells.^29^, ^30^, ^31^ Exosomes can serve as important components of extracellular vesicles (EVs) and are abundant in the extracellular matrix, reflecting and affecting the property of MSCs.^32^, ^33^ Other than directly participating in tissue regeneration, reports suggest that the effect of stem cells is also attributed to the release of cytokines and EVs.^34^, ^35^ Previous studies have indicated that exosomes derived from clinical‐grade oral mucosal epithelial cell sheets could inhibit fibroblast proliferation and promote growth factor gene expression, showing pro‐regenerative effects on skin wound healing.^36^ Here, we explored whether exosomes derived from SHED aggregates would promote SHED aggregate‐based pulp regeneration. We found that SA‐Exo were necessary for neovascularization and pulp tissue regeneration. Moreover, the combined use of SHED aggregate and SA‐Exo would significantly promote neovascularization and pulp tissue regeneration.

Angiogenesis is essential for tissue repair and regeneration as blood vessels support cells with nutrition and oxygen.^37^ MSCs are considered as a pharmacological and therapeutic approach to accelerate angiogenesis, including endothelial differentiation and bioactive molecule secretion.^38^ Recently, exosomes derived from MSCs have been reported to play an effective role in angiogenesis regulation.^39^, ^40^, ^41^ As for pulp regeneration, neovascularization of the regenerated pulp tissue was demonstrated not only derived from host endothelial cells but also from SHED aggregate implantation.^10^ It has been validated that SHED are capable of differentiating into HUVECs and secreted factors from SHED can directly activate HUVECs to promote all processes of angiogenesis.^12^, ^13^, ^42^, ^43^ Given the crucial role of SA‐Exo in pulp tissue regeneration in the animal study, we are interested in how SA‐Exo promotes angiogenesis after SHED aggregate implantation. We found that SA‐Exo significantly promoted SHED themself endothelial differentiation and upregulated endothelial cell function, which may contribute to vascularization thus supporting tissue regeneration.

Exosomes encapsulate and convey various bioactive cargos that are further transmitted to neighbouring or distant cells, where they induce various signalling cascades.^32^ As an important component of exosomal cargos, miRNAs are implicated in modulating endothelial cell function and angiogenesis.^44^, ^45^ Therefore, to investigate the mechanisms of SA‐Exo in pulp regeneration, we analysed the miRNA expression profile of SA‐Exo and found that miR‐26a was markedly upregulated in SA‐Exo. MiR‐26a belongs to the microRNA‐26 family, which can be induced by hypoxia and upregulated during multiple cell differentiation, participating in the development of normal tissues and many pathological processes.^46^ It has been reported that miR‐26a plays an effective role in bone regeneration through positive regulation of angiogenic‐osteogenic coupling. Moreover, exosomal miR‐26a can promote angiogenesis of microvessel HUVECs in glioma.^47^ In addition, glucocorticoid‐induced osteonecrosis of the femoral head can be prevented by exosomal miR‐26a by promoting angiogenesis and osteogenesis.^48^ However, another study demonstrated that miR‐26a is highly expressed in the endothelium and negatively regulates the BMP/SMAD1 signalling axis, thereby inhibiting endothelial proliferation and angiogenesis.^49^ Here, we found that miR‐26a contributed to the proangiogenic role of SA‐Exo by promoting SHED endothelial differentiation and enhancing endothelial cell function, indicating the role of SHED aggregate‐derived miR‐26a in angiogenesis and its potential use in the pulp or other tissue regeneration.

The miRNAs are involved in the highly complex network of cell signalling pathways, which play important roles in various cellular processes, including proliferation, differentiation, apoptosis, development and so on.^50^ In this study, signalling analysis showed that the TGF‐β pathway was highly expressed in SA‐Exo. As is well‐known, the TGF‐β signalling pathway plays a critical role in regulating vascular function in health and disease,^51^ while multiple TGF‐β signalling components can be modulated by miRNAs.^52^ TGF‐β/SMAD2/3 signalling pathway plays important role in endothelial mesenchymal transformation and angiogenesis. Previous study indicates that miRNA‐26a can target the 3'UTRs of TGF‐βR2 and regulate scar hypertrophy by mediating TGFβR1/2.^53^ Moreover, miR‐26a can promote the expression of related genes in the TGF‐β/SMAD pathway.^54^ In addition, miR‐26b has been reported to impede Stanford type A aortic dissection (TAAD) development by regulating its direct target genes HMGA2 and TGF‐β/Smad3 signalling pathway.^55^ According to our cell experiments, we found that SMAD2/3, TGFβ RI and TGFβ RII can be upregulated by miR‐26a, which can be downregulated by miR‐26a inhibition. Moreover, the proangiogenic effects of SA‐Exo‐derived miR‐26a on SHED and HUVECs can be ameliorated by using a TGFβ RI inhibitor. These data further demonstrated that miR‐26a participates in the modulation of TGF‐β signalling, thereby promoting SHED endothelial differentiation and endothelial cell function. Additional animal experiments are required to further examine whether miR‐26a contributes to SA‐Exo‐mediated angiogenesis and pulp regeneration *via* TGF‐β/ SMAD2/3 signalling in vivo.

In summary, our study revealed the underlying mechanisms of the SHED aggregate involved in pulp regeneration, which was closely related to SA‐Exo‐derived miR‐26a conducing to angiogenesis *via* TGF‐β/SMAD2/3 signalling. Our study provides new evidence to expand the possibility of SA‐Exo as a new strategy to promote angiogenesis and pulp tissue regeneration.

## CONFLICT OF INTEREST

The authors have no conflicts of interest to declare.

## AUTHOR CONTRIBUTIONS

Meiling Wu, Xuemei Liu and Zihan Li contributed to the study design, manuscript preparation and data collection. Xiaoyao Huang and Hao Guo contributed to the analysis of in vitro study. Xiaohe Guo and Xiaoxue Yang contributed to animal experiments. Bei Li, Kun Xuan and Yan Jin developed the concept, supervised the research, and critically revised the manuscript. All authors contributed to the article and approved the final manuscript.

## Data Availability

The data sets in this study are available from the corresponding author on reasonable request.

## APPENDIX 1

### MATERIALS AND METHODS

#### Transwell assay

Cell migration was assessed by a Transwell assay using an 8 μm pore transwell insert (Corning, United States). 2 × 10^4^ SHED or HUVECs were seeded into the upper chamber in a serum‐free medium. The lower chamber was filled with 500 μL of conditioned medium. After culturing for 6 h, the migrated cells were fixed for 15 min and stained with crystal violet for 10 min. The number of the migrating cells was determined as the mean of cell number in five random fields under the microscope.

#### Quantitative real‐time polymerase chain reaction (qRT‐PCR)

The relative gene expression of angiogenesis genes was tested by qRT‐PCR. Total RNA was extracted using the TRIzol Reagent (Accurate Biotechnology, AG21101). Then, cDNA was synthesized from 1 μg total RNA using Prime Script RT Master Mix (Takara, Japan). The qRT‐PCR analyses were performed on CFX96 TouchTM (BIO‐RAD, United States) using TB GreenTM Premix EX TaqTM II (Takara, Japan). GAPDH was used as an internal control for normalization. The primers used are listed below:

**Table:**

qRT‐PCR Primer Sequences			
Gene	Sequences		
*VEGFA*		F:AGGGCAGAATCATCACGAAGT	R:AGGGTCTCGATTGGATGGCA
*PDGFA*		F:GCAAGACCAGGACGGTCATTT	R:GGCACTTGACACTGCTCGT
*Angiopoitein*		F:CTGGGCGTTTTGTTGTTGGTC	R:GGTTTGGCATCATAGTGCTGG

#### Western blot analysis

Cells and exosomes were lysed with RIPA (Beyotime, China) supplemented with protease inhibitor (04693132001, Roche, United States) on ice. The BCA was used to perform protein quantification. 20 μg of the extracted protein was electrophoresed on SDS polyacrylamide gel electrophoresis (PAGE) and transferred onto polyvinylidene fluoride (PVDF) membranes (Roche). After blocking with 5% bovine serum albumin (MP Biomedicals) for 1 h, the membranes were incubated using primary antibodies overnight at 4℃, followed by incubation with appropriate secondary antibodies. Finally, blots were visualized with an enhanced chemiluminescence system using the Tanon Chemidoc Apparatus (Tanon‐Bio, China) and quantified using ImageJ software. The primary antibodies used were anti‐angiopoietin 2 antibody (ab155106, Abcam), PDGF (3174, Cell Signaling Technology, United States), VEGF (ab46154, Abcam), P‐SMAD2/3 (sc‐11769, Santa Cruz Biotechnology, United States), SMAD2/3 (ab202445, Abcam), TGFβ RI (sc‐398, Santa Cruz Biotechnology), TGFβ RII (sc‐400, Santa Cruz Biotechnology) and GAPDH (30201ES20, YEASEN, China).
